# Between Two Worlds: Investigating the Intersection of Human Expertise and Machine Learning in the Case of Coronary Artery Disease Diagnosis

**DOI:** 10.3390/bioengineering11100957

**Published:** 2024-09-25

**Authors:** Ioannis D. Apostolopoulos, Nikolaos I. Papandrianos, Dimitrios J. Apostolopoulos, Elpiniki Papageorgiou

**Affiliations:** 1 Department of Energy Systems, University of Thessaly, Gaiopolis Campus, 41500 Larisa, Greece; ece7216@upnet.gr (I.D.A.); npapandrianos@uth.gr (N.I.P.); 2 Department of Nuclear Medicine, University Hospital of Patras, 26504 Rio, Greece; dimap@med.upatras.gr

**Keywords:** coronary artery disease, machine learning, random forest, probability calibration

## Abstract

Coronary artery disease (CAD) presents a significant global health burden, with early and accurate diagnostics crucial for effective management and treatment strategies. This study evaluates the efficacy of human evaluators compared to a Random Forest (RF) machine learning model in predicting CAD risk. It investigates the impact of incorporating human clinical judgments into the RF model’s predictive capabilities. We recruited 606 patients from the Department of Nuclear Medicine at the University Hospital of Patras, Greece, from 16 February 2018 to 28 February 2022. Clinical data inputs included age, sex, comprehensive cardiovascular history (including prior myocardial infarction and revascularisation), CAD predisposing factors (such as hypertension, dyslipidemia, smoking, diabetes, and peripheral arteriopathy), baseline ECG abnormalities, and symptomatic descriptions ranging from asymptomatic states to angina-like symptoms and dyspnea on exertion. The diagnostic accuracies of human evaluators and the RF model (when trained with datasets inclusive of human judges’ assessments) were comparable at 79% and 80.17%, respectively. However, the performance of the RF model notably declined to 73.76% when human clinical judgments were excluded from its training dataset. These results highlight a potential synergistic relationship between human expertise and advanced algorithmic predictions, suggesting a hybrid approach as a promising direction for enhancing CAD diagnostics.

## 1. Introduction

Coronary artery disease (CAD) remains a challenge to contemporary healthcare systems, substantially contributing to global mortality rates [1,2]. Despite significant advances in diagnostic methods, the non-trivial nature of CAD complications and the inherent complexity accompanying its diagnosis pose difficulties [2,3]. Interpreting diagnostic results is frequently a delicate and challenging task involving several potential parameters and factors, such as variant symptomatology and imaging artefacts [4]. Acknowledging such ambiguity articulates the critical need to elevate the quality of diagnostic precision in CAD detection.

Subsequently, emerging approaches primarily bolstered by advancements in artificial intelligence (AI), machine learning (ML), and deep learning (DL) signify a paradigm shift in diagnostic methodologies [4,5,6,7]. AI, ML, and DL engines analyze extensive datasets from various clinical parameters and imaging technologies to uncover subtle anomalies and patterns, potentially accelerating the CAD diagnosis process or providing a helpful decision support tool [8]. These advancements use modern computational tools to complement clinical decisions, offering diagnostic precision.

However, despite their transformative capacity, AI, ML, and DL-enhanced diagnostic tools are not exempt from constraints. Key among these is the propensity toward dataset bias, compromising algorithm effectiveness due to skewed or non-representative datasets [8,9]. Moreover, the black-box algorithms exhibiting an opaque decision-making process may hinder acceptance and trust from clinicians [10]. Furthermore, the overreliance on retrospective data could hamper the peripheral application of these AI tools in real-life clinical settings, necessitating continuous validation and re-assessment.

Several research studies propose multiple ML and DL models for diagnosing CAD non-invasively via automated processing of imaging modalities, such as SPECT [11,12,13,14,15,16,17,18,19,20,21,22,23,24]. Many studies have also dealt with automatic identification of CAD using non-imaging data, such as demographic and clinical information, symptoms and other diagnostic tests [23,24,25,26,27,28,29,30,31,32,33]. Recently, many studies have proposed the synergistic use of MPI and clinical data for holistic CAD diagnosis approaches with better results [23,34,35,36,37].

Much discussion is held around the role of human experts in such AI systems [25]. A striking lack of studies assessing concurrence between AI-determined diagnoses and clinician-provided diagnoses compounds the challenge. Consequentially, omitting clinician’s experiential wisdom from the AI framework could deprive the systems of invaluable insights, further undermining the joint synergy of human expertise and computational power. The necessity of conflating the clinician’s wisdom into the diagnostic process and AI systems remains undisputed, making it an imperative area for technological development.

This study seeks to further examine and quantify the integration of human expert evaluations with ML in the context of CAD risk assessment. Building on the existing literature, our research suggests that diagnostic accuracy and reliability improvements can be achieved via this integration. We hypothesize that an ML model enriched with human evaluative inputs will demonstrate superior performance metrics compared to their sole data-driven counterparts. We utilized a comprehensive dataset that includes typical risk factors associated with CAD and human expert evaluations of these factors to examine the synergistic effects on an ML model’s performance. The novelty of our study lies in its investigation into the synergistic integration of human expert evaluations with machine learning (ML) models in the context of coronary artery disease (CAD) risk assessment. Unlike previous studies focusing solely on either data-driven algorithms or clinical expertise, this research uniquely combines both to enhance diagnostic precision and reliability. We provide empirical evidence that incorporating human judgments significantly boosts the performance of ML models, achieving higher accuracy than models relying only on objective data.

## 2. Materials and Methods

### 2.1. Research Methodology

**Figure 1:**
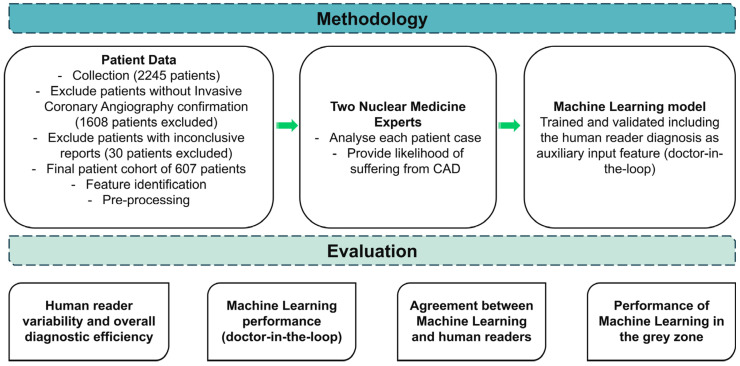
Research methodology.

An overview of the research methodology is illustrated in Figure 1. The research leveraged a dataset encompassing CAD risk factors, which is vital for a practical diagnostic evaluation. Distinctive variables such as gender, age, family history of heart disease, presence of diabetes, and Electrocardiogram (ECG) test results stood as the dominant features in the dataset (Section 2.2). The definitive diagnosis (either non-CAD or CAD), ascertained through rigorous medical examinations and consistent clinical follow-up, was included.

Τwo impartial human evaluators (N.P. and D.J.A. of the authors), who were rendered unaware of the actual patient condition, attributed their subjective judgements regarding the potentiality of each case to CAD disease. Using a six-tier scale, which spanned from “Highly Unlikely” to “Highly Likely”, the human readers assigned a likelihood score to each case based on their discernment of the risk factors. This process yielded a thorough understanding of the diagnostic efficacy of human specialists in evaluating CAD risks.

The ML model was trained and evaluated based on the annotated dataset of CAD characteristics, both with and without including the evaluators’ judgments as additional features.

Firstly, the diagnostic efficacy of human experts was intensively analyzed to comprehend their capability to predict CAD risk. Key performance metrics like accuracy, sensitivity, specificity, and the Area Under the Receiver Operating Characteristic Curve (AUC-ROC) were computed to assess the reliability of human evaluators in performing such a task.

Following the detailed human analysis, the ML model was evaluated independently. The model was initially trained merely on the CAD-related features, excluding human evaluators’ judgments. This evaluation aimed to understand the model’s potential to predict CAD accurately without considering subjective clinical judgments.

The model was reassessed after integrating human evaluators’ scores as additional features into the training dataset. This aimed to delineate the influence of incorporating human evaluators’ subjective judgments on model performance. By considering human specialist insights alongside objective CAD risk factors, the model was anticipated to refine their predictions, potentially elevating the diagnostic accuracy and confidence level.

Moreover, the correlation between ML probability predictions and human evaluators’ scores was critically examined to evaluate the convergence of digital and clinical assessments. This analysis aimed to determine whether the ML models can mirror human specialists’ judgments, shedding light on the potential synergies that could arise from combining automated algorithms and human intuition in medical decision-making.

Lastly, cases that fell within ambiguous zones—those characterized by neither distinctly high nor low probabilities—were thoroughly analyzed. We isolated patients falling into “Slightly likely” or “Slightly unlikely” by either human reader, creating a grey zone where diagnostic certainty was ambiguous. This grey zone represents a critical area in medical diagnosis where clinical decisions become challenging. Next, we analyzed the best human reader’s and ML’s performance (using the expert’s diagnostic yield as an additional input feature) on these cases. This evaluation aimed to assess the discriminative ability of the ML model under uncertain clinical situations, contributing insights into their performance when faced with diagnostic challenges akin to those encountered in CAD management. Through these comprehensive evaluations, the research methodology sought to extract the potential synergistic attributes of ML and human proficiency in enhancing diagnostic accuracy and confidence in CAD risk characterization.

### 2.2. Coronary Artery Disease

CAD is the most common type of heart disease and a leading cause of death worldwide [1]. It occurs when the coronary arteries that supply blood to the heart muscle become hardened and narrowed due to the buildup of cholesterol and other materials, known as plaque, on their inner walls [2]. This process, known as atherosclerosis, can reduce blood flow to the heart, leading to symptoms such as chest pain (angina), shortness of breath, and other cardiac problems. Acute blockage of these arteries can cause a myocardial infarction or heart attack, posing severe health risks and requiring immediate medical attention.

Diagnosing CAD effectively is critical for preventing severe cardiovascular events. While invasive methods like invasive coronary angiography (ICA) are considered gold standards for diagnosing CAD, they are not without risks, such as bleeding, infections, and arterial damage. Consequently, there is significant interest in reliable, non-invasive diagnostic techniques. However, these non-invasive methods come with their own set of challenges. CAD exhibits a range of symptoms from severe, clear-cut chest pain to subtle or no symptoms, especially at the early stages or in atypical patient populations such as women and diabetic patients. Commonly used non-invasive tests include electrocardiograms, stress tests, and echocardiography. These can often result in ambiguous outcomes. For instance, imaging methods can be limited by the image’s resolution, the patient’s body habitus, and the interpreter’s experience [38], leading to sub-optimal diagnostic performance.

ML offers promising avenues to enhance the diagnosis of CAD through non-invasive means. By integrating and analyzing clinical data, ML models can uncover complex patterns that might not be readily visible to human evaluators [39]. ML algorithms can combine diverse data types (clinical data, patient history, basic test results) more comprehensively than traditional statistical methods. The latter can lead to improved predictive accuracy for CAD, helping in early and more accurate diagnosis.

Once trained and validated, ML models can be deployed relatively easily across various platforms, including in regions that lack the infrastructure for advanced imaging technologies. ML can also augment the capabilities of existing imaging techniques. By applying advanced image processing algorithms, such as deep learning, to interpret images, the accuracy of non-invasive imaging methods for detecting CAD can be significantly enhanced.

### 2.3. Patient Cohort

The research used patient data recorded in the Department of Nuclear Medicine of the University Hospital of Patras, Greece, from 16 February 2018 to 28 February 2022. Over this period, 2245 patients underwent gated-SPECT MPI with ^99m^Tc-tetrofosmin. Two-hybrid SPECT/CT gamma-camera systems (Varicam, Hawkeye and Infinia, Hawkey-4, GE Healthcare, Chicago, IL, USA) were used for Myocardial Perfusion Imaging (MPI). The Ethical Committee of the abovementioned hospital approved the study. The nature of the survey waives the requirement to obtain patients’ informed consent. Table 1 summarizes the CAD risk factors and includes a range of predisposing factors, recurrent diseases, symptoms, and diagnostic test results. The clinical characteristics of the dataset are presented in Table 2. No missing values were reported during the data collection process.

MPI interpretation and reporting was carried out prospectively and independently by two experienced Nuclear Medicine physicians (DJA and NP). The interpreters inspected the complete set of tomographic slices and the polar maps. Their final report has also considered all pertinent clinical information, such as detailed patient history, symptoms, CAD predisposing factors, previous test results, baseline ECG and ECG changes during stress, etc. The two nuclear medicine experts reviewed patients with suspected CAD from the dataset. Considering all risk factors, they independently assigned risk scores using a scale from one to six (with one representing the highly unlikely probability of suffering from CAD and six representing the highly likely probability).

### 2.4. Data Pre-Processing

Data pre-processing is essential in any ML workflow. Pre-processing helps improve the model’s performance and ensures that the input data are correctly and meaningfully represented. This section outlines the specific pre-processing steps undertaken for the various features involved in this study.

Several features in the dataset are categorical, including the patient’s gender, presence of known CAD, previous AMI (Acute Myocardial Infarction), previous PCI (Percutaneous Coronary Intervention), previous CABG (Coronary Artery Bypass Graft), prior stroke, presence of diabetes, smoking habits, arterial hypertension, dyslipidemia, peripheral angiopathy, chronic kidney disease, and family history of CAD. Moreover, potential symptoms are categorized as asymptomatic, atypical angina, angina-like events, dyspnoea on exertion, and incidents of precordial chest pain. The expert’s opinion is also present in the dataset. These categorical variables contain classes that are nominal or ordinal and, hence, require appropriate encoding.

For binary categorical features such as gender, known CAD, diabetes, and more, binary encoding will be applied, where ‘0’ and ‘1’ denote a condition’s absence or presence, respectively. One-hot encoding is utilized for multi-class categorical features like expert’s opinion (which can vary between highly unlikely, unlikely, slightly unlikely, sightly likely, likely, and highly likely). This approach transforms each categorical value into a new column and assigns a 1 or 0 (True/False).

Numerical features such as age and body mass weight (BMI) are crucial for accurately predicting CAD. However, their range can vary significantly, possibly biasing an ML model. To counteract this, standardization (or Z-score normalization) is applied. This technique involves subtracting the mean and dividing it by the standard deviation for each value of a numerical feature, transforming them to have zero mean and a standard deviation of one. This scaling ensures that each feature contributes equally to the model’s prediction.

### 2.5. Machine Learning

#### 2.5.1. Random Forest

The RF algorithm is an ensemble learning method that operates by constructing many decision trees from a dataset and yielding the class output based on the mode of the classes for classification tasks. At its core, the algorithm harmonizes the power of multiple decision trees to refine the final decision, thereby creating a “forest”. The RF algorithm was selected for diagnosing CAD due to its performance in similar tasks [34,40].

In terms of implementation, the RandomForestClassifier was invoked with parameters max_depth = 5, min_samples_leaf = 1, min_samples_split = 2, and n_estimators = 100. These parameters fine-tune the behaviour of the RF algorithm, according to a grid search approach, which exhaustively searches among several parameter and hyper-parameter combinations to define the optimal. The max_depth parameter limits the maximum depth of the tree to prevent overfitting. Min_samples_leaf is the minimum number of samples required to constitute a leaf node, which prevents the creation of overly complex trees. Min_samples_split is the minimum number of samples needed to split an internal node. The n_estimators parameter, on the other hand, signifies the number of trees in the forest, set at 100 in this instance.

Building decision trees begins by randomly selecting a subset of features and data points from the dataset. It then identifies the best feature among the subset to split the data based on specific criteria (impurity), forming the tree’s root. This process is repeated to produce the next split (node), and so forth, until the tree reaches its maximum permitted depth or can no longer split due to lack of samples. Once all the trees are constructed, the RF algorithm predicts by letting each tree in the forest vote for a class, and the class with the majority of votes is selected as the final output.

RF minimizes overfitting by averaging the results of its decision trees, which increases the algorithm’s robustness and generalization capability. By considering the votes from all decision trees, the RF model offers a more reliable and comprehensive means of diagnosing CAD, consequently outperforming a stand-alone decision tree model.

#### 2.5.2. Probability Calibration

The necessity for probability calibration arises from the intrinsic disparity often observed between the raw probability outputs of a classifier, like the RF employed in this study, and the actual observed frequencies. Specifically, in the domain of CAD diagnosis, it is paramount that the probability outputs representing diagnostic predictions are realistic and interpretable to healthcare professionals. This is particularly critical when integrating ML insights with expert judgments, as excessively high or low probability values could mislead or cause hesitancy in clinical decision-making.

In this context, while the RF model offers promising classification abilities, its probability estimates for the occurrence of a disease like CAD are not always well-calibrated. This misalignment can be attributed to the model’s nature of aggregation and voting from multiple trees, where probabilities are generally pushed towards extreme values (close to 0 or 1). In the particular dataset, the initial RF probability scores for each class are given in Figure S1. Therefore, the primary goal of probability calibration is to adjust these raw probabilities to represent true likelihoods as closely as possible, aligning them more effectively with the expert-provided probability scores.

The calibration approach adopted in this study involves the application of a post-processing technique known as sigmoid calibration or Platt Scaling. This method uses logistic regression to map the uncalibrated probabilities to calibrated probabilities. The process involves fitting a logistic regression model on the output of the base classifier (the RF model in this case) to learn a transformation that maps the original probabilities to the calibrated scale. Platt Scaling transforms the output scores (f) of the RF classifier into calibrated probabilities (p) using logistic regression. The transformation can be modelled mathematically as:

$$P\left(y=1|x\right)=\frac{1}{1+e^{\left(Af\left(x\right)+B\right)}}$$

where *A* and *B* are parameters learned by fitting the logistic regression to a dataset where *f*(*x*) are the outputs of the classifier and the targets are the true class labels (0 or 1 in binary classification tasks like CAD diagnosis). The parameters *A* and *B* are estimated using maximum likelihood estimation.

To implement this, we utilized the CalibratedClassifierCV module from Sklearn’s Python library with the ‘sigmoid’ method, ensuring that the original decision function of the classifier is retained and only probabilities are adjusted. Notably, the procedure includes training the calibrator on the same training data used for the RF model to maintain consistency and relevance of calibration.

The effectiveness of this calibration can be assessed by comparing the calibration curves before and after applying the Platt Scaling and by evaluating using the Expected Calibration Error (*ECE*), which is given below:

$$ECE=\sum_{m=1}^{M}\frac{\left|B_{m}\right|}{n}|acc\left(B_{m}\right)-conf(B_{m})|$$

*ECE* splits the data into *M* equally spaced bins. *B* is used to represent each bean (*m* is the bin number). The expression *acc(B_m_)* is the accuracy within the bin *B_m_*, defined as the average of the true outcomes for the samples in the bin. The expression *conf(B_m_)* is the confidence (mean predicted probability) within *B_m_*.

Moreover, additional testing with the expert assessments and their provided probabilities will validate how well the calibrated probabilities align with professional judgment.

By calibrating the probability outputs of the RF model, the results can be expected to yield more practical and reliable diagnostic probabilities.

We conducted our experiments using Python 3.9, leveraging essential libraries such as Numpy for numerical computations, Pandas for data manipulation, Scikit-Learn for machine learning algorithms, and Statsmodels for statistics.

## 3. Results

### 3.1. Patient Analysis

Table 2 presents the analysis of the study’s dataset. Of the 2245 patients that underwent gated-SPECT MPI with ^99m^Tc-tetrofosmin, six hundred thirty-seven (28.8%) were subsequently subjected to invasive coronary angiography (ICA) within 60 days from MPI for further evaluation. Thirty patients were excluded from the dataset due to inconclusive MPI results or missing ICA reports.

Invasive Coronary Angiography obtained the actual patient’s condition (label). Invasive Coronary Angiography was considered positive for flow-limiting CAD if lesions causing >50% lumen stenosis of the left main artery or >70% stenosis of the major coronary artery branches were identified. Fractional flow reserve (FFR) measurements were undertaken in case of intermediate lesions (causing 50–70% stenosis). An FFR value <0.8 was considered positive for flow-limiting CAD in such instances.

### 3.2. Human Reader Diagnostic Performance

Table 3 presents the distribution of scores assigned by the two readers among the potential risk categories.

The first reader classified 343 patients in lower risk categories (highly unlikely, likely, slightly unlikely), and 264 patients are the rest. The second reader classified 336 patients in lower risk categories and 271 in higher risk categories. Variability between the medical experts was observed and was reflected in the diagnostic performance of each one. Specifically, the first reader had 203 true positives, 61 false positives, 279 true negatives, and 64 false negatives (sensitivity = 76%, specificity = 82%). The second reader had 204 true positives, 67 false positives, 273 true negatives, and 63 false negatives (sensitivity = 76.4%, specificity = 80%). The two readers performed similarly in the binary classification problem (CAD, Healthy), with a mean accuracy of 79%. Considering ambiguous patient cases falling into the categories of slightly likely or slightly unlikely, the first expert assigned 243 patients into the grey zone, while the second expert assigned 224.

**Figure 2:**
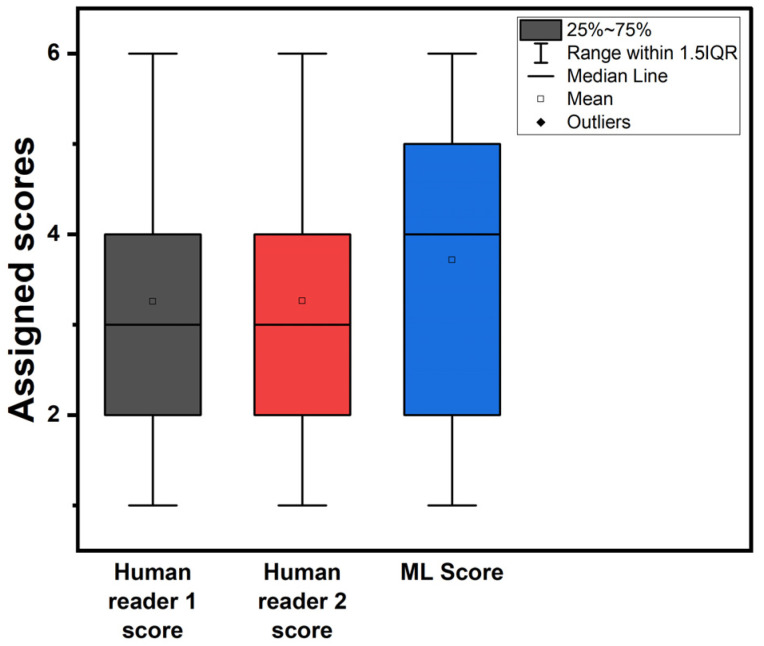
Box plots of assigned probability categories. ML score refers to the RF model trained with the experts’ diagnosis as an additional feature.

**Figure 3:**
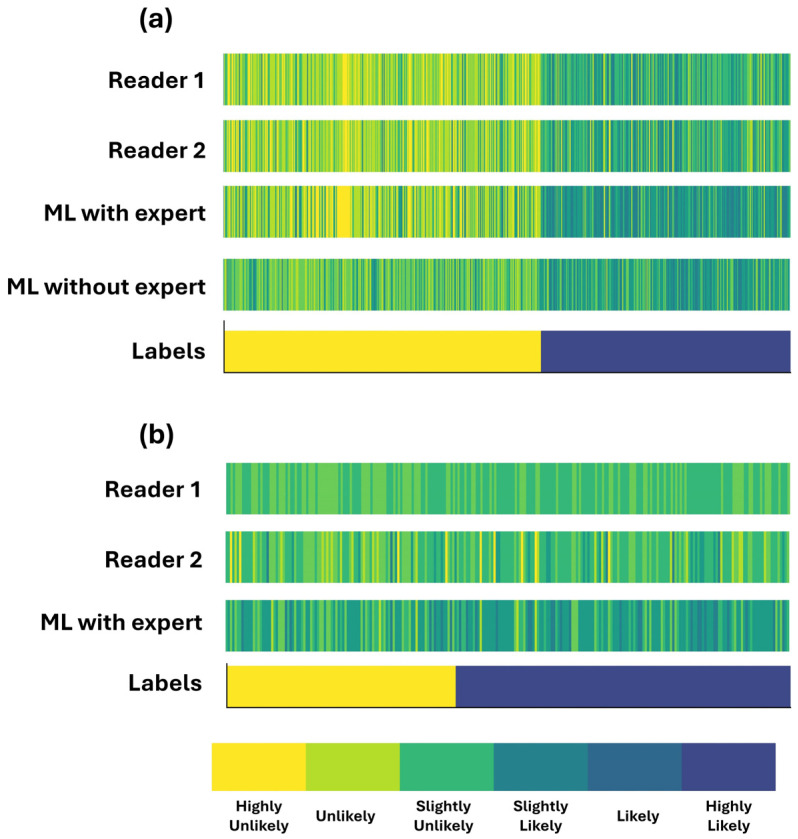
Assigned CAD-probability categories per patient case and their true labels. Patient cases are sorted by label across the *x*-axis. (**a**) complete dataset (**b**) grey zone.

Figure 2 illustrates box plots for the assigned probability categories among patients by the two readers and the RF model when using the expert’s diagnosis as an additional input feature. Figure 3 shows the assigned CAD-probability categories per patient case and their true labels among all classification methods.

The discrepancy between the two readers is observed in neighbouring categories (e.g., an expert assigns a patient as “Likely”, whereas the second reader classifies them as “Slightly likely”). The latter conclusion can be observed in Figure 3a.

### 3.3. Human-Assisted ML Performance

RF was trained and validated following a 10-fold cross-validation procedure. Besides the risk-related objective features, the expert’s diagnostic yield was also supplied as an input feature to the ML model. Table 4 provides the evaluation metrics.

RF correctly identified 221 CAD patients (TP) and 265 healthy (TN). FP cases were 75, and FN cases were 46. Subsequently, the model’s accuracy reached 0.8017 (CI 95%: 0.8–0.8034). The model’s sensitivity is 0.8286 (95% CI: 0.8256–0.8315), suggesting that it correctly identifies TP in 82.86% of cases. Specificity, which measures the ability to correctly identify TN, is 0.7805 (95% CI: 0.7786–0.7825). The F1 score, a balance between precision and recall, is 0.7861 (95% CI: 0.7842–0.788), indicating good overall performance.

The area under the receiver operating characteristic curve is 0.8917 (95% CI: 0.8914–0.8921), indicating high discriminative ability. Regarding actual counts, there are 221 TP, 265 TN, 75 FP, and 46 FN. The false positive rate (FPR) is 0.2195 (95% CI: 0.2175–0.2214), and FNR is 0.1714 (95% CI: 0.1685–0.1744). PPV is 0.7478 (95% CI: 0.746–0.7496), indicating the proportion of true positive predictions, and the NPV is 0.8529 (95% CI: 0.8507–0.8551), indicating the proportion of true negative predictions. These results collectively suggest that the model can discriminate between classes and performs well in terms of sensitivity and specificity.

### 3.4. Impact of Experts’ Diagnostic Yield on the Performance of ML

RF was trained and evaluated, excluding the medical experts’ diagnosis from the input features. Table 5 presents the performance of the RF algorithm without the experts’ diagnostic yield as an additional input feature.

The model achieved an accuracy of 80.17% with the additional input feature, significantly outperforming the model without it, which had an accuracy of 73.76%. This indicates the importance of including expert knowledge in the model, as it improves its ability to correctly classify instances.

Furthermore, the sensitivity of the RF model when using the expert’s assessment is notably higher (82.86%) compared to training without it (65.09%). This means that when experts’ diagnostic yield is included, the model is better at identifying true positives, which are instances of the condition being correctly classified. This improvement in sensitivity suggests that the model with the additional input feature is more adept at capturing the positive instances accurately, which is crucial in medical diagnosis or any scenario where correctly identifying positives is vital.

On the other hand, the specificity of the RF model is slightly lower (78.05%) compared to Table 5 (80.57%). This indicates that the model without the additional input feature is marginally better at identifying true negatives, instances where the condition is correctly identified as absent. However, this small decrease in specificity is outweighed by the significant increase in sensitivity, resulting in a higher overall accuracy.

The F1 score, which combines precision and recall, is substantially higher in Table 4 (78.61%) compared to Table 5 (68.58%). This suggests that the model’s ability to balance precision and recall is better when experts’ diagnostic yield is included, indicating more robust performance overall.

**Figure 4:**
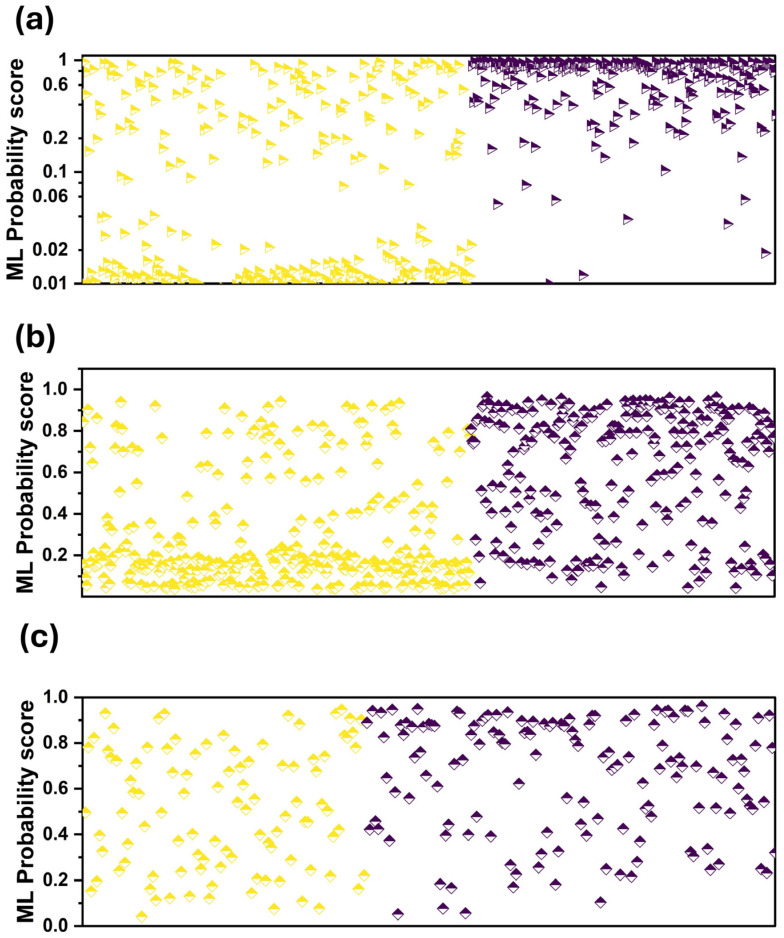
Probability scores of ML per patient case. Purple colour: CAD, yellow: Healthy. (**a**) when using the expert’s diagnosis as an additional input feature, (**b**) without using the expert’s diagnosis as an additional input feature, (**c**) grey zone and when using the expert’s diagnosis as an additional input feature.

We observed that the assigned probability scores are better aligned with those designated by the readers (Figure 3a). This is also verified by the two scatter plots of Figure 4a,b. Figure 4a shows that RF is more confident in its predictions, assigning low probability scores between zero and 0.02 and high probability scores between 0.6 and one.

### 3.5. Grey Zone Results

Table 6 presents the diagnostic performance of the best human reader and the ML model in ambiguous cases (grey zone).

ML performed similarly to the best human reader (reader’s accuracy = 0.6219, ML accuracy = 0.6341). However, we observed variability in their decisions per patient, which is reflected in the sensitivity and specificity scores. ML had better sensitivity (0.87) than the reader (0.6712), whereas the reader had better specificity (0.55 versus 0.29). ML had fewer FN instances (19) compared to the reader (48) and more FP instances (71 versus 45). The latter observation suggests that ML may be beneficial in ambiguous cases because of the low FN numbers. FN is considered a more serious mistake than FP in the case of CAD. An FP diagnosis subjects the patient to ICA, the ultimate diagnosis and treatment of CAD. On the contrary, an FN diagnosis suggests no further action, though necessary. Therefore, especially in the grey zone, a model with larger sensitivity and specificity may be preferable to a model with a balanced trade-off.

### 3.6. Feature Importance

RF was utilized to identify the relative importance of various features. The significance assigned to each feature by the RF model provides insights into their relative contributions towards diagnosing CAD (Table S1). The most influential feature is “Human judgement”, which is valued at 22%, underscoring the critical role of clinical expertise and assessment in CAD diagnosis despite advancements in automated diagnostic tools. This suggests that while statistical models and patient data are invaluable, the subjective insights of healthcare professionals remain paramount.

The next significant feature is “Atypical chest pain”, marked at 11% importance. This emphasizes the diagnostic challenge in cases where symptoms do not conform strictly to typical CAD manifestations, requiring careful interpretation and consideration by clinicians.

Other symptoms related to exertion, such as “Dyspnea on Exertion”, “Angina-like”, and “Gender” each hold a 6% importance. This reflects an understanding that CAD often affects physiological responses to physical stress, and symptoms exhibited can substantially guide diagnosis.

“Age” and “Diabetes” are also notable, each with 7% and 6% importance, respectively. Age is a well-established risk factor in CAD, influencing the likelihood of its occurrence. Diabetes is recognized for its significant impact on cardiovascular health, leading to increased risk of CAD amongst diabetic patients.

Conversely, factors such as “Previous myocardial infarction”, “Previous revascularisation (PCI and CABG)”, “Previous stroke”, and “End-stage renal failure” show surprisingly low importance values (all at 1%). This suggests that while these factors are critical in a patient’s medical history, they may not independently alter the RF model’s prediction of current CAD status as much as the symptomatic and judgment-based assessments.

## 4. Discussion

The primary aim of this study was to assess ML performance in diagnosing CAD compared to human evaluators’ performance in assigning CAD risk scores and the extent to which incorporating human-annotated risk scores into the model could improve its performance.

The human evaluators’ CAD risk assessments yielded an average accuracy of 79%, while the RF ML model, incorporating human risk assessments, produced an accuracy of 80.17%. These findings indicate that the ML model and human evaluators had comparable efficacy in predicting CAD risk. Both demonstrated the capability to confidently assign CAD risk categories, having implications on how CAD could be effectively managed.

Although the discrepancy between the human readers was noted, particularly regarding adjacent patient categories, a promising outcome became evident when these human assessments were combined with the RF model. An uptick in accuracy, sensitivity, and specificity was observed following the inclusion of human evaluators’ scores in the training dataset, reinforcing the hypothesis that human intuition complements the ML model’s data-driven assessments.

Interestingly, the ML model’s performance diminished when run independently without considering the evaluators’ judgments, reducing accuracy to 73.76%. This implies that the evaluators’ subjective judgments hold diagnostic value beyond mere supplementary information. The latter is also verified by the fact that AI managed to increase the overall accuracy by approximately 1% and only when using human judgement as an additional input. They enrich the objective risk factor data, providing a more accurate CAD risk prediction. Hence, it highlights the value of a symbiotic relationship between ML algorithms and human expertise in medical diagnostics.

When considering the ambiguous cases, or the ‘grey zone’, adding human evaluators’ scores enhanced the ML model’s discriminative ability amidst uncertain clinical situations. Incorporating human specialist insights equips the ML model with robust tools to tackle diagnostic challenges akin to those faced in CAD management, manifesting the cooperative potential of ML and human evaluations.

Central to the successful implementation of our ML model in clinical settings is the role of human clinicians, who serve not merely as end-users but as active participants in a man-in-the-loop system. This approach facilitates a symbiotic interaction where the ML model augments the clinicians’ diagnostic capabilities while clinicians provide continual input to refine and guide the model’s performance. In practice, this dynamic would see the ML model generating preliminary risk assessments based on patient data, which clinicians then review and potentially adjust before a final decision is made.

Furthermore, this man-in-the-loop approach may offer several advantages, including mitigating potential biases inherent in the data used by the ML model. By involving clinicians actively, the system learns from these human insights, reducing over-reliance on purely data-driven algorithms that may overlook subtleties in patient symptoms or histories.

### Limitations

While the promising outcomes underpin the potential synergies between ML algorithms and human evaluations, further investigation is necessary to validate these findings in various contexts and with different ML frameworks.

One significant limitation is that the study results are specifically derived from a population undergoing invasive CAD testing and may not be generalizable to other populations. This restricts the applicability of the findings to only similar clinical settings, limiting wider extrapolation to those not screened for CAD in the same manner. The study’s dataset comprised only 607 patients collected from a single hospital in Greece. This relatively small sample size is less than ideal for developing and validating robust models. Typically, larger datasets, potentially gathered from varying demographics and multiple centres, are preferable to enhance the model’s generalizability and to ensure that it captures a broader spectrum of patient presentations and outcomes.

Moreover, the study’s reliance on a single-centre design further constrains the generalizability of the findings. Data from a single hospital may not represent the variability found across different geographic locations and healthcare systems.

The validation process of the model also presents limitations. Cross-validation within the same dataset may not adequately represent the model’s performance in real-world settings.

Additionally, the integration of expert ratings in the study was handled by adding these as an additional feature in the model. Exploring more sophisticated methods for integrating these sources of information could potentially enhance predictive performance and clinical relevance. Also, having the same experts provide data for model inputs and serve as the benchmark for human predictions introduces potential bias, which might affect the validity of the results.

Lastly, the model’s reliance on a limited set of clinical variables is a potential drawback. Incorporating a more comprehensive array of data types, such as imaging results, genomic information, or longer-term patient outcomes, could enrich the model’s dataset. This, in turn, may enhance the model’s ability to predict more accurately and adapt to the nuances of individual patient cases.

## 5. Conclusions

This study illustrates the feasible combination of ML algorithms and human expertise to enhance the predictability and reliability of CAD risk assessments. It demonstrated that the human evaluators and the RF model had comparable efficacy in predicting CAD risk. Increased accuracy was observed when evaluators’ judgments were incorporated, illuminating their essential diagnostic value in enriching traditional CAD risk factors. Consequently, a significant decline in ML model performance was noted when human judgments were excluded, emphasizing their critical role in overall diagnostic accuracy. Incorporating human specialist insights improved the ML model’s diagnostic ability under uncertain situations, underlining the potential cooperative benefits. These outcomes underscore the potential synergy between ML algorithms and human evaluations.

## Figures and Tables

**Table 1:** Dataset’s attributes and potential values.

Attribute Name	Value Range
Gender	Male/Female
Age	Numeric
BMI	Numeric
History of known CAD	Yes/No
Previous myocardial infarction	Yes/No
Previous revascularisation PCI	Yes/No
Previous revascularisation CABG	Yes/No
Previous stroke	Yes/No
Diabetes	Yes/No
Smoking	Yes/No
Hypertension	Yes/No
Dislipidemia	Yes/No
Peripheral arteriopathy	Yes/No
End-stage renal failure	Yes/No
Family History of premature CAD	Yes/No
Previous ETT	Normal/Abnormal
Asymptomatic	Yes/No
Atypical chest pain	Yes/No
Angina-like	Yes/No
Dyspnea on Exertion	Yes/No
Incident of chest pain	Yes/No
Baseline ECG	Normal/Abnormal
Human judgement	highly unlikely, unlikely, slightly unlikely, sightly likely, likely, and highly likely

*PCI: percutaneous coronary intervention; CABG: coronary artery by-pass grafting; BMI: Body Mass Index; ETT: exercise treadmill test.*

**Table 2:** Patient characteristics.

Clinical Characteristics	Frequency
No	607
Age (mean ± sd)	67 ± 10 years
Sex (male/female)	77%/23%
History of CAD	32%
Previous myocardial infarction Previous revascularisation (PCI/CABG) *	21% 22%
Previous stroke	1%
Hypertension	77%
Dyslipidemia	65%
Smoking	39%
Diabetes	27%
Peripheral arteriopathy	0.5%
End-stage renal failure	0.5%
Family history of premature CAD	17%
Abnormal baseline ECG **	33%
Symptoms	?
Asymptomatic	44%
Atypical chest pain	15%
Angina-like symptoms	13%
Incident of chest pain (with the subsequent biochemical exclusion of acute coronary syndrome)	12%
Dyspnea on exertion	16%

* PCI: percutaneous coronary intervention; CABG: coronary artery by-pass grafting; ** *ECG: electrocardiogram.*

**Table 3:** Distribution of human reader assignments among the potential classes.

Score Groups	Reader 1	Reader 2
Highly unlikely	51	68
Unlikely	189	174
Slightly unlikely	103	94
Slightly likely	143	130
Likely	60	83
Highly unlikely	61	58

**Table 4:** RF diagnostic performance using the experts’ diagnostic yield as an additional input feature.

Evaluation Metric	Value
Accuracy	0.8017 (CI 95%: 0.8–0.8034)
Sensitivity	0.8286 (CI 95%: 0.8256–0.8315)
Specificity	0.7805 (CI 95%: 0.7786–0.7825)
F1	0.7861 (CI 95%: 0.7842–0.788)
AUC	0.8917 (CI 95%: 0.8914–0.8921)
TP	221
TN	265
FP	75
FN	46
FPR	0.2195 (CI 95%: 0.2175–0.2214)
FNR	0.1714 (CI 95%: 0.1685–0.1744)
PPV	0.7478 (CI 95%: 0.746–0.7496)
NPV	0.8529 (CI 95%: 0.8507–0.8551)

**Table 5:** RF diagnostic performance without the experts’ diagnostic yield as an additional input feature.

Evaluation Metric	Value
Accuracy	0.7376 (CI 95%: 0.736–0.7392)
Sensitivity	0.6509 (CI 95%: 0.6481–0.6538)
Specificity	0.8057 (CI 95%: 0.8019–0.8095)
F1	0.6858 (CI 95%: 0.6842–0.6873)
AUC	0.8039 (CI 95%: 0.8026–0.8052)
TP	174
TN	274
FP	66
FN	93
FPR	0.1943 (CI 95%: 0.1905–0.1981)
FNR	0.3491 (CI 95%: 0.3462–0.3519)
PPV	0.7247 (CI 95%: 0.7213–0.7281)
NPV	0.7462 (CI 95%: 0.745–0.7473)

**Table 6:** Performance of the best reader and ML in cases considered ambiguous by the two readers.

Evaluation Metric	Best Human Reader	ML
Accuracy	0.6219	0.6341
Sensitivity	0.6712	0.87
Specificity	0.55	0.29
TP	98	127
TN	55	29
FP	45	71
FN	48	19

## Data Availability

The datasets analyzed during the current study are available from the nuclear medicine physician upon reasonable request.

## Supplementary Materials

The following supporting information can be downloaded at: https://www.mdpi.com/article/10.3390/bioengineering11100957/s1, Figure S1: Initial probabilites of the RF model using the human judgement as an additional input feature. Red triangles represent the Healthy class and blue represent the CAD class; Table S1: Feature Importance.
