# Supplementary material for: Between Two Worlds: Investigating the Intersection of Human Expertise and Machine Learning in the Case of Coronary Artery Disease Diagnosis
Source: Bioengineering (Basel). 2024 Sep 25;11(10):957. doi: 10.3390/bioengineering11100957 (PMC11504143; doi:10.3390/bioengineering11100957)
Supplement: Supplementary file 1 [file bioengineering-11-00957-s001.zip › bioengineering-3177246-supplementary.pdf]

bioengineering

MDPI

Article

Between Two Worlds: Investigating the Intersection of Human Expertise and Machine Learning in the Case of Coronary Artery Disease Diagnosis

Supplementary Materials

1. Evaluation metrics

We used many evaluation metrics commonly used in similar binary classification problems in the medical domain. Accuracy, which measures the proportion of correctly classified instances, provides a basic overview of model correctness. Sensitivity, also known as the True Positive Rate (TPR), indicates the model's ability to correctly identify positive cases among all actual positives, thus emphasizing its capability to detect medical significance. Conversely, Specificity, or the True Negative Rate (TNR), showcases the model's skill in avoiding false alarms for negative cases, an essential aspect in medical diagnostics to prevent unnecessary interventions. Precision, or Positive Predictive Value (PPV), assesses the proportion of true positive predictions among all positive predictions, offering insights into the model's capability to avoid false positives, which is particularly crucial in medical contexts where misdiagnosis can lead to serious consequences. The F1 score, a harmonic mean of precision and recall, balances these two measures, providing a single value that indicates overall performance. Area Under the ROC Curve (AUC-ROC) quantifies the model's ability to discriminate between positive and negative classes across different thresholds, offering a comprehensive measure of discriminative power. True Positives (TP) represent the number of correctly classified positive instances, while True Negatives (TN) denote correctly classified negative instances. False Positives (FP) and False Negatives (FN) are the numbers of incorrectly classified positive and negative instances, respectively. False Positive Rate (FPR) and False Negative Rate (FNR) represent the proportions of incorrectly classified negative and positive instances, respectively. Positive Predictive Value (PPV) and Negative Predictive Value (NPV) indicate the proportions of true positive and true negative predictions, respectively, among all predictions.

The abovementioned metrics are derived from the equations below:

$$Accuracy = \frac{TP+TN}{TP+TN+FP+FN} \quad (1)$$

$$Sensitivity = \frac{TP}{TP+FN} \quad (2)$$

$$Specificity = \frac{TN}{TN+FP} \quad (3)$$

$$False\ Positive\ Rate = \frac{FP}{TN+FP} \quad (4)$$

$$False\ Negative\ Rate = \frac{FN}{TP+FN} \quad (5)$$

$$Positive\ Predictive\ Value = \frac{TP}{TP+FP} \quad (6)$$

$$Negative\ Predictive\ Value = \frac{TN}{TN+FN} \quad (7)$$

2. Feature Importance

Table S1. Feature Importance

Input Feature	Importance (%)
Gender	6
Age	7
BMI	5
History of known CAD	1
Previous myocardial infarction	1
Previous revascularisation PCI	2
Previous revascularisation CABG	1
Previous stroke	1
Diabetes	6
Smoking	4
Hypertension	2
Dislipidemia	1
Peripheral arteriopathy	1
End-stage renal failure	1
Family History of premature CAD	2
Previous ETT	2
Asymptomatic	5
Atypical chest pain	11
Angina-like	6
Dyspnea on Exertion	6
Incident of chest pain	5
Baseline ECG	2
Human judgement	22

3. Probability calibration

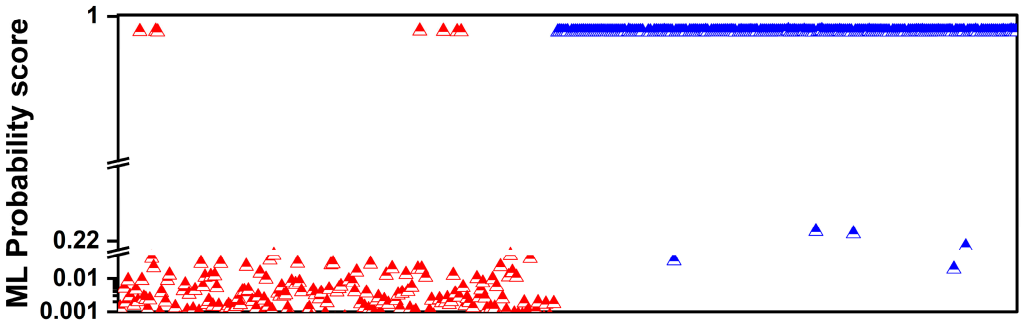

Figure S1. Initial probabilites of the RF model using the human judgement as an additional input feature. Red triangles represent the Healthy class and blue represent the CAD class.
